# Retraction: Molecular predictors of brain metastasis-related microRNAs in lung adenocarcinoma

**DOI:** 10.1371/journal.pgen.1010552

**Published:** 2022-12-16

**Authors:** The PLOS Genetics Editors

Following publication of this article [1], concerns were raised about some of the microscopy images in Figs 4, 5, and 7, and about the size of the tumors in the experiments reported in the article.

Specifically,

- The H157-NC Migration panel in Fig 4C of [1] appears similar to the mimic+ WNT5A plasmid Migration panel in Fig 5d of [2].
- The A973-NC Invasion panel in Fig 5C of [1] appears similar to the A549-NC Migration panel in Fig 2H of [3].
- The control mimic + control plasmid Migration panel and the mimic+P21 plasmid Migration panel in Fig 7F were incorrectly duplicated. A revised Fig 7 was provided in a Correction [4] to address the duplication.
- The tumor volume data reported in Figs 2 and 3 indicate that tumor volumes in some animals may have exceeded internationally-accepted standards for humane endpoint limits for mouse tumor studies.
- The underlying microarray data for Fig 1 were not deposited in a public repository at the time of publication; however, upon editorial follow up the authors have deposited the microarray data in Gene Expression Omnibus (GEO) under accession GSE186666, resolving this concern.

The first author contacted the journal editors to report an error in the charts shown in Figs 2C and 3C, and provided revised tumor volume charts reporting smaller tumor volumes compared to the published figures. The first author stated that vernier calipers were used to measure tumor volume, and that the errors in the published tumor volume data were found during routine raw data inspection. The underlying individual-level quantitative data for all figures were provided, along with the *in vivo* imaging system (IVIS) output files underlying the photographs in Figs 2C and 3C and all experimental replicates, and a copy of the ethical approval document with reference number NCC2014GZ-01.

Editorial review of the underlying tumor volume data and IVIS data identified potential inconsistencies such that concerns about the reliability of the tumor volume data could not be fully resolved.

In light of the concerns about the above image issues and about the reliability of the tumor volume data, which could not be fully resolved by the underlying data provided, the *PLOS Genetics* Editors retract this article.

GS, XDing, NB, ZW, LWu, WZhou, ZZ, JW, Weimin Zhang, JF, WenJue Zhang, XDong, YS, QZ and LWang agreed with the retraction, stand by the article’s findings, and apologize for the issues with the published article. NL either did not respond directly or could not be reached.
